# High-yield BMP2 expression in rice cells *via* CRISPR and endogenous *αAmy3* promoter

**DOI:** 10.1007/s00253-024-13054-0

**Published:** 2024-02-14

**Authors:** Thi Mai Nguyen, Pei-Yi Wu, Chih-Hung Chang, Li-Fen Huang

**Affiliations:** 1 Graduate School of Biotechnology and Bioengineering, Yuan Ze University, Taoyuan City, 320 Taiwan, Republic of China; 2 Department of Life Sciences, National Central University, Taoyuan City, 320 Taiwan, Republic of China; 3 Department of Orthopedic Surgery, Far Eastern Memorial Hospital, New Taipei City, Taiwan, Republic of China

**Keywords:** CRISPR/Cas 9 knock-in, Rice suspension cells, Sugar, *αAmy3* promoter, Human BMP2 recombinant protein

## Abstract

**Abstract:**

Plant cells serve as versatile platforms for the production of high-value recombinant proteins. This study explored the efficacy of utilizing an endogenous *αAmy3* promoter for the expression of a bioactive pharmaceutical protein, specifically the mature region of human bone morphogenetic protein 2 (hBMP2m). Utilizing a refined CRISPR/Cas9-mediated intron-targeting insertion technique, which incorporates an artificial 3’ splicing site upstream of the target gene, we achieved a transformation efficiency of 13.5% in rice calli that carried the rice-codon optimized mature region of *hBMP2* cDNA (*rhBMP2m*) in the *αAmy3* intron 1. Both homozygous and heterozygous *rhBMP2m* knock-in rice suspension cell lines were generated. These lines demonstrated the endogenous *αAmy3* promoter regulated *rhBMP2m* mRNA and rhBMP2m recombinant protein expression, with strongly upregulation in respond to sugar depletion. The homozygous *rhBMP2m* knock-in cell line yielded an impressive 21.5 μg/mL of rhBMP2m recombinant protein, accounting for 1.03% of the total soluble protein. The high-yield expression was stably maintained across two generations, indicating the genetic stability of rhBMP2m gene knock-in at the *αAmy3* intron 1 locus. Additionally, the rice cell-derived rhBMP2m proteins were found to be glycosylated, capable of dimer formation, and bioactive. Our results indicate that the endogenous rice *αAmy3* promoter–signal peptide-based expression system is an effective strategy for producing bioactive pharmaceutical proteins.

**Key points:**

• *The endogenous αAmy3 promoter-based expression system enhanced the yield of BMP2*

• *The increased yield of BMP2 accounted for 1.03% of the total rice-soluble proteins*

• *The rice-produced BMP2 showed glycosylation modifications, dimer formation, and bioactivity*

**Supplementary information:**

The online version contains supplementary material available at 10.1007/s00253-024-13054-0.

## Introduction

Advances in genetic engineering have facilitated successful production of recombinant proteins in various host systems (Ma et al. 2003). To ensure optimal biological activity and stability of recombinant proteins through post-translational modifications, higher eukaryotic cells are often required as host cells to produce recombinant biopharmaceutical proteins (Shanmugaraj et al. 2020). As higher eukaryotic cells, plant cells possess intrinsic capabilities for post-translational protein modifications. Moreover, plant cells offer distinct advantages of low production costs and high biosafety of the resulting products (Ghag et al. 2021; Singh et al. 2021). Consequently, plant cells have emerged as an up-and-coming alternative for producing valuable biopharmaceutical recombinant proteins such as antibodies, antigens, and cytokines (Daniell et al. 2009).

The *αAmy3* (or *αAmy3D*) promoter-based recombinant production system is widely recognized and extensively used to produce recombinant proteins (Chi and Huang 2022; Kuo et al. 2013). This system utilizes the strong induction of *αAmy3* promoter in response to sugar starvation (Lu et al. 1998). In addition, the system enables the secretion of recombinant proteins directly into the liquid culture medium by incorporating the αAmy3 signal peptide (Chen et al. 2004). An expression cassette is typically constructed by integrating a recombinant gene with the *αAmy3* promoter, a signal peptide, and a transcription terminator. This expression cassette was subsequently introduced into rice cells using Agrobacterium- or particle bombardment-mediated transformation to initiate protein expression. However, the transgene expression levels may vary attributed to random integration events and differing copy numbers within the rice genome, resulting in unpredictable phenotypes in transgene expression (Hong et al. 2006; Kim et al. 2008).

The CRISPR/Cas9 system is a potent tool for the precise genetic modification of target genes within the genome. This system induces DNA double-strand breaks at predetermined sites in the genome (Cong et al. 2013), and the subsequent gene knock-out or knock-in processes at the target location follow two DNA repair mechanisms: non-homologous end-joining (NHEJ) and homologous recombination (HR). NHEJ often leads to changes in base pairs or insertion/deletion of fragments (Haque et al. 2018; Ma et al. 2015). Conversely, HR uses long homologous donor DNA fragments at the target locus to replace the desired DNA region, thereby facilitating DNA fragment knock-in. Although HR-mediated gene editing is used for targeted DNA fragment knock-in in plants, it generally exhibits low efficiency (Huang and Puchta 2019; Li et al. 2018; Sun et al. 2016). Recently, an alternative approach has been developed to achieve DNA fragment knock-in in plants via NHEJ repair mechanism (Dong and Ronald 2021; Dong et al. 2020; Li et al. 2016; Xu et al. 2020). This breakthrough highlighted the successful gene knock-ins at specific locations in the plant genome.

Previously, we established a recombinant protein expression system using the endogenous *αAmy3* promoter and a signal peptide to express GFP reporter proteins in rice suspension cultures (Nguyen et al. 2022). The *GFP* gene was regulated by the endogenous *αAmy3* promoter; therefore, it exhibited intense expression under sugar-deficient conditions. Furthermore, the resulting GFP recombinant proteins were efficiently secreted into the culture medium, which was mediated by the αAmy3 signal peptide.

Bone morphogenetic protein 2 (BMP2), a member of the transforming growth factor β (TGF-β) superfamily, plays crucial roles particularly in bone development, osteoblast differentiation, and fracture repair. Clinical applications of BMP2 include bone regeneration and repair, spinal fusion, dental implants, orthopedic surgery, and tissue engineering (Halloran et al. 2020). BMP2 is initially synthesized as an inactive precursor pre-pro-hBMP-2, which undergoes post-translational modifications to form an active mature hBMP2 homodimer with an approximate molecular weight of 30 kDa (Heng et al. 2010; Wozney et al. 1988). Each monomer consists of 114 amino acids and includes an N-glycosylation site (Israel et al. 1992). Currently, the production of recombinant human BMP2 (rhBMP2) relies on Chinese hamster ovary (CHO) cells and *E. coli* (Israel et al. 1992; Kubler et al. 1998; Vallejo et al. 2002). However, these commercial expression systems encounter difficult challenges such as a lack of protein post-translational modifications, incorrect protein folding, low protein stability, high production costs, and the risk of potential human–pathogen contamination (Bessho et al. 2000; Demain and Vaishnav 2009; Park et al. 2014).

Various plant-based production systems, such as tobacco leaves and soybean seeds, have successfully produced hBMP2 (Ceresoli et al. 2016; Queiroz et al. 2019; Rahimifard Hamedani et al. 2021; Suo et al. 2006). However, these systems typically only produce pro-hBMP2 or β-glucuronidase-fused, γ-zein-fused, or hydrophobin-fused hBMP2, and yield of the mature hBMP2 (hBMP2m) in current plant systems is low. For example, in transgenic tobacco leaves, fusing hBMP2m with GUS and zein at the N-terminus resulted in a low yield of 0.02% TSP of hBMP2m recombinant protein after removing the fused domains (Ceresoli et al. 2016; Suo et al. 2006). This low yield highlights a significant limitation in the efficiency of plant cells for the production of hBMP2m. In this study, we used an endogenous *αAmy3* promoter–signal peptide-based recombinant protein expression system to produce hBMP2m in rice suspension cultures. We conducted codon optimization of the mature human BMP2 cDNA (*rhBMP2m*) in rice and successfully inserted it into intron 1 of *αAmy3*. Heterozygous and homozygous *rhBMP2m* cDNA knock-in rice suspension cell lines were obtained from the T0 and T1 generation. The homozygous *rhBMP2m* knock-in suspension cell line showed high expression of *rhBMP2m* mRNA and recombinant rhBMP2m protein (rhBMP2m). Furthermore, we analyzed the characteristics of the rhBMP2m protein, including dimer formation, N-glycosylation, protein yield, and bioactivity.

## Materials and methods

### Plant materials

*Oryza sativa* L. cv. Tainung No. 67 (TNG67) was used in this study. Immature seeds of TNG67 were sterilized, and plated on CIM1 agar medium, which comprised MS medium supplemented with vitamins (Duchefa, Haarlem, Netherlands), 0.03% Casamino acid, 0.2% L-proline, 10 μM 2,4-dichlorophenoxyacetic acid (2,4-D), 3% sucrose, and 0.4% phytagel to generate embryonic calli, and incubated at 28 °C under constant light (Sinaga et al. 2021). Embryonic calli were derived from the scutellum and sub-cultured on fresh CIM1 agar medium for gene transformation. The calli were transferred to N6 liquid medium containing 10 μM 2,4-D and 3% sucrose to establish cell suspension cultures, as described previously (Nguyen et al. 2022). The suspension-cultured cells were maintained at 28 °C on an orbital shaker at 110 rpm in a dark culture room and subcultured in fresh N6 liquid medium every week.

### Plasmid and plasmid construction

The CRISPR plasmid, C3sgRNA-Cas9, was constructed to accommodate a gRNA expression cassette that targets the *αAmy3* intron I, as described in (Nguyen et al. 2022). The *rhBMP2m* donor plasmid was constructed by synthesizing the rice codon-optimized *rhBMP2m* and subcloning it into the master donor plasmid, pET28a-C3gRNA, at *Asc*I and *Not*I sites to generate the pC3gRNA-rhBMP2 donor plasmid.

### Plant transformation

The C3sgRNA-Cas9 and pC3gRNA-rhBMP2 plasmids were combined at a molar ratio of 1:3. Subsequently, 1.5 mg of 1.0 μm gold particles were coated with 10 μg of the mixed plasmid DNA. The immature embryo-derived calli were transferred into half-strength Murashige and Skoog medium supplemented with 0.4% phytagel and bombarded once with 500 μg DNA-coated gold particles using a PDS-1000/He particle delivery system (Bio-Rad, Hercules, CA, USA) at 1100 psi with a target distance of 9 cm as previously described (Lu et al. 2002). Transformed calli were selected on N6 medium containing 50 mg/L hygromycin B as previously described (Lu et al. 2002). Hygromycin-resistant calli were used to regenerate T0 transgenic rice plants on a regeneration medium (Ozawa et al. 2003). These T0 plants were then self-pollinated in a greenhouse to produce T1 seeds. The T1 seeds were subsequently used to induce T1 calli and establish suspension cell cultures.

### PCR-based genotype analysis

Genomic DNA was extracted from calli using a previously reported method (Huang et al. 2020). Briefly, rice samples were ground with liquid nitrogen using a mortar and pestle, and genomic DNA was extracted using an extraction buffer containing 100 mM Tris-HCl (pH 8.0), 50 mM EDTA (pH 8.0), 100 mM NaCl, 1% SDS, and 1% β-mercaptoethanol. Subsequently, 1 μg of each genomic DNA sample was amplified via PCR with various gene-specific primers (Supplemental Table S1). Finally, the resulting PCR products were separated via electrophoresis on 1% agarose gel.

### RT-PCR

Total RNA was isolated from calli using a previously described method (Sinaga et al. 2021). Briefly, rice cell samples were ground with liquid nitrogen using a mortar and pestle. The powdered sample was then transferred into a pre-chilled Eppendorf tube, and total RNA was extracted using TRIzol Reagent (Invitrogen, Carlsbad, CA, USA) according to the manufacturer’s protocol. DNase I (Invitrogen) treatment was performed to eliminate any possible DNA contamination. Subsequently, first-strand cDNA was synthesized using SuperScript III Reverse Transcriptase (Invitrogen), 2.5 µg of total RNA, and oligo-dT primers. A tenfold dilution of the resulting first-strand cDNA was amplified via PCR with gene-specific primers for *ACT1*, *rhBMP2m*, *αAmy3*, and *αAmy3-rhBMP2m* (Supplemental Table S1). PCR was conducted using Taq DNA polymerase (Invitrogen) in a thermal cycler (Biomatra, Göttingen, Germany). PCR was amplified 26 cycles for *ACT1* and *αAmy3*, and 28 cycles for *rhBMP2m* and *αAmy3-rhBMP2m*. Each cycle consisted of denaturation at 95 °C for 20 s, annealing at 50 °C for 45 s, and extension at 72 °C for 1 min. The PCR products were separated using electrophoresis on a 1% agarose gel.

### Western blot analysis

Protein gel blot analysis was performed as previously described (Nguyen et al. 2022). Total soluble cellular protein was extracted from the rice suspension cells, and cell-culture medium samples were collected to detect secretory proteins. Protein samples were separated by SDS-PAGE and transferred onto PVDF membranes. The primary antibodies used were polyclonal rabbit anti-human BMP2 (Abcam, Cambridge, MA, USA) and αAmy3 (Agrisera, Vännäs, Sweden) antibodies at a 1:1000 dilution and 1:2000 dilution, respectively. HRP-conjugated anti-rabbit IgG was used as the secondary antibody to detect rhBMP2m and αAmy3 proteins. Target protein signals were detected through chemiluminescence using an ECL prime western blotting detection kit (GE Healthcare, Chicago, IL, USA).

### Enzyme-linked immunosorbent assay (ELISA)

The concentration of rhBMP2m in rice suspension cultures was determined by ELISA following the method described by Liu (Liu et al. 2012).

### Alkaline phosphatase (ALP) activity assay

The bioactivity of rBMP2m was analyzed by the alkaline phosphatase activity assay system (ab83369, Abcam). Briefly, C2C12 cells plated in 96-well plates (1 × 10^4^/well) were treated with indicated concentrations of rBMP2m in 100 μl Dulbecco's Modified Eagle Medium (DMEM) supplemented with 2% Fetal Bovine Serum (FBS). After 72 h of incubation, the culture medium was removed, followed by a PBS wash. Then, 50 μl ALP buffer was added to each well, and the plate was frozen at -80 °C. After two freeze-and-thaw cycles, 50 μl 5 mM p-nitrophenylphosphate (pNPP) solution was added to each well. The plate was incubated at RT for 60 min, protected from light. Twenty microliters of stop solution were added to each well, and the absorbance of 405 nm was measured by the Cytation 7 cell imaging multimode reader (BioTek, Santa Clara, CA, USA).

## Results

### Generation of CRISPR-Cas 9-engineered rice cells with human *BMP2m* cDNA insertion at the intron I of *αAmy3*

**Fig. 1:**
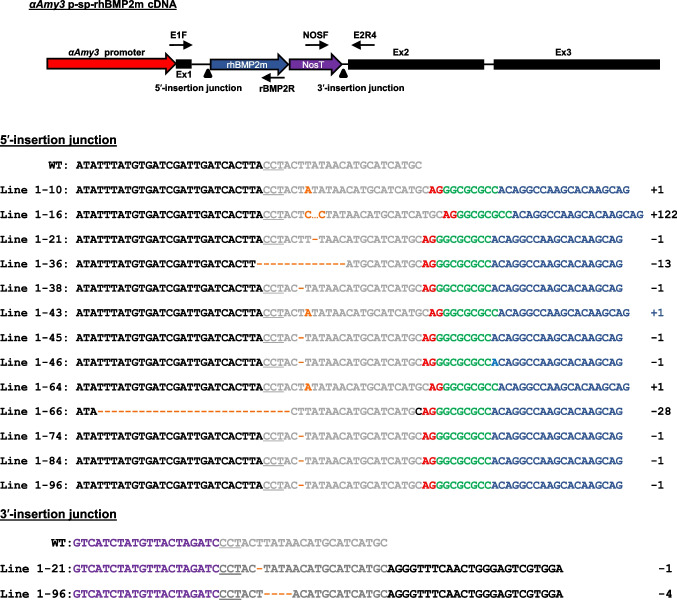
Results of the DNA sequencing of the *rhBMP2m* insertion junction at the target site of the *αAmy3* intron I in rice cell lines. DNA fragments containing the 5′- and 3′-insertion junctions were amplified from various transgenic rice cell lines using specific primer pairs: E1F and rBMP2R primers for the 5′ junction, and NOSF and E2R4 primers for the 3′ junction. The positions of these primers are indicated on a map of the *αAmy3p-sp-rhBMP2m* DNA. The amplified DNA fragments were then subjected to DNA sequencing. For the results of the 5′ junction sequencing, black characters represent rice genomic DNA sequences upstream of the C3 site, gray characters represent sequences of C3 site, underlined indicates PAM sites, red characters represent artificial 3′ splicing site, green characters represent *Not*I restriction enzyme adaptor, and blue characters represent *rhBMP2m* DNA sequences. Regarding the 3′ junction sequencing results, purple characters represent nos terminator sequences (*nos*T), gray characters represent C3 site sequences, and black characters represent exon 2 sequences. Orange characters and hyphens within C3 site indicate inserted and deleted nucleotides, respectively. Negative (–) and positive ( +) signs indicate the number of nucleotides deleted and inserted at the intron-target site. The wild-type is represented by "WT," whereas the line number indicates the knock-in cell lines

In a previous study (Nguyen et al. 2022), we established an expression system based on the endogenous rice *αAmy3* promoter and a signal peptide using a modified CRISPR/Cas9 knock-in approach. To produce rhBMP2m in rice suspension cells, we co-transformed a CRISPR plasmid containing an *αAmy3*-targeted gRNA module and a donor plasmid containing an artificial 3' splice site upstream of the rice-codon-optimized mature human *BMP2* cDNA (*rhBMP2m*) (Supplementary Fig. S1) into the immature rice embryogenic calli via particle bombardment. Ninety-six hygromycin-resistant calli were obtained in this study. Subsequent genotype detection using E1F and rBMP2R primers (Supplementary Table 1) and PCR-based analysis revealed that 13 calli harbored potential *αAmy3SP*-*rhBMP2m* DNA fragments (Supplementary Fig. S2). To analyze the insertion site of the 13 putative lines, we conducted a 5'-junction sequence analysis. The *rhBMP2m* cDNA fragments were found to follow the *αAmy3* intron DNA sequence, and different junction sequences were identified in individual rice calli (Fig. 1; Supplementary Fig. S3). To validate the *rhBMP2m* cDNA insertion patterns, we selected two cell lines, Line-21 and Line-96, and analyzed the 3' junctions of *rhBMP2m* cDNA insertion site. The amplified DNA fragment sequence confirmed that the *Nos* terminator DNA region (nosT) was located within the *αAmy3* intron 1 (Fig. 1). These results indicate that all independent lines harbored the *rhBMP2m* cDNA integrated at intron 1 of *αAmy3* with a forward orientation. The efficiency of the CRISPR/Cas9 knock-in approach for inserting the *rhBMP2m* cDNA fragment into intron 1 of *αAmy3* was 13.5%.

### Endogenous *αAmy3* promoter regulates the expression of mature human BMP2 recombinant protein in rice cells

**Fig. 2:**
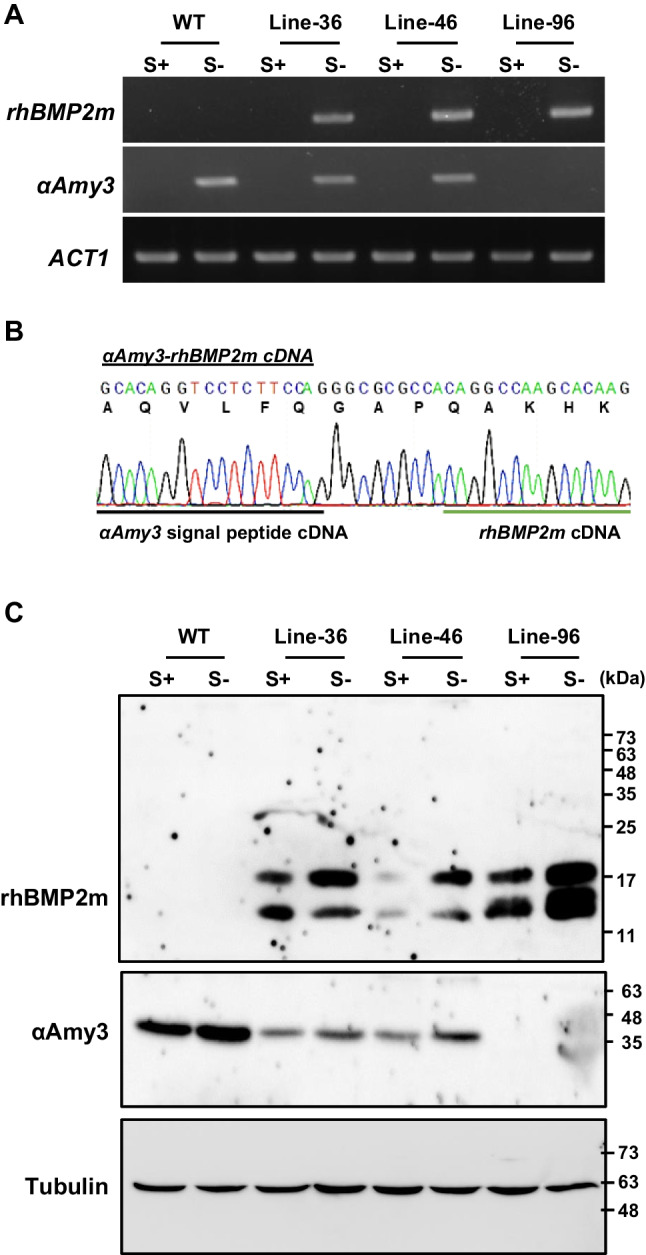
Expression of recombinant *rhBMP2m* in knock-in rice cell lines. (**A**) Detection of *rhBMP2m* mRNA in knock-in rice suspension cell lines. Total RNAs were isolated from wild-type (WT) and three knock-in suspension cell lines (Lines-36, -46, and -96) after 18 h of culture in a sugar-fed (S +) or sugar-starved (S-) media. RNAs underwent RT-PCR analysis with *αAmy3-rhBMP*2*m*, *αAmy3*, and *ACT1* specific primers (Table S1). (**B**) DNA sequencing result for the *αAmy3-rhBMP2m* cDNA. The *αAmy3-rhBMP2m* cDNA was synthesized by RT-PCR using total RNAs from Line-96. The black underline indicates the *αAmy3* signal peptide cDNA, whereas the blue underline characterizes the *rhBMP2m* cDNA. The deduced amino acids of the *αAmy3-rhBMP2m* fusion protein are shown at the top of the panel. (**C**) Detection of recombinant human BMP2 protein (rhBMP2m) in knock-in rice suspension cells. Following eight days of culture in both sugar-fed (S +) and sugar-starved (S-) media, total soluble proteins were isolated from WT, Line-36, Line-46, and Line-96 cells. Western blot analysis was conducted using human BMP2, αAmy3, and tubulin antibodies. The molecular weight markers, in kDa, are represented on the left side of the figure

To establish suspension cell lines, three independent *rhBMP2m* knock-in cell lines were selected, namely Lines-36, -46, and -96. Genotype analysis revealed that the Lines-36 and -46 suspension cultures contained both *αAmy3SP-rhBMP2m* and endogenous *αAmy3* DNA fragments, whereas the suspension cells of Line-96 contained only *αAmy3SP-rhBMP2m* DNA fragments (Supplementary Fig. S4). This result suggests that Line-96 represents as a homozygous *αAmy3SP-rhBMP2m* cell line. The endogenous *αAmy3* promoter in cultured rice suspension cells is known to be activated by sugar depletion (Lu et al. 1998). To investigate whether the endogenous *αAmy3* promoter regulates the transcription of *rhBMP2m* gene in these knock-in cell lines, total RNA was extracted from sugar-fed and sugar-starved cells after 18 h and subjected to RT-PCR analysis. The results showed that *rhBMP2m* mRNAs were detected in all selected sugar-starved cells (Fig. 2A), indicating that the inserted *rhBMP2m* gene was expressed and controlled by the endogenous *αAmy3* promoter. High levels of endogenous *αAmy3* mRNA were observed in sugar-starved cells of Lines-36 and -46, similar to wild-type cells, but not in Line-96 (Fig. 2A). These results indicated that all selected cell lines were responsive to sugar signals and provided further evidence to support the conclusion that Line-96 is a homozygous *rhBMP2m* knock-in cell line.

To further investigate whether the newly created intron was removed from the *αAmy3SP-rhBMP2m* pre-mRNA, the *αAmy3SP-rhBMP2m* cDNA region was amplified from Line-96 using RT-PCR and subjected to DNA sequencing. The *αAmy3* signal peptide region of the cDNA was confirmed to be fused in-frame upstream of the *rhBMP2m* cDNA, indicating that the intron was correctly spliced out from the *αAmy3SP-rhBMP2m* pre-mRNA (Fig. 2B).

The production of rhBMP2m recombinant protein was analyzed in *rhBMP2m* knock-in cell lines by western blotting with specific human BMP2 antibodies. Cell suspensions from Lines-36, -46, and -96 with cell densities of 24% (cell volume/medium volume) were cultured in sugar-containing and sugar-free media for eight days. Two specific bands with molecular weights of approximately 14 and 18 kDa of rhBMP2m proteins were detected in the sugar-starved cells of all the selected cell lines (Fig. 2C). Liu et al. previously reported that, at an initial density of 24% (v/v) rice suspension cells cultured in a sugar-containing medium, the sugar concentration dropped rapidly and almost no sugar was detected on day 7 (Liu et al. 2012). Thus, relatively low levels of rhBMP2m protein were detected in the cell suspension initially incubated in sugar-containing medium for 8 days (Fig. 2C). The abundance of rhBMP2m was the highest in Line-96, followed by Lines-36 and -46, regardless of whether the cells were cultured with or without sugar medium. Tubulin was used as an internal control for equal loading evaluations (Fig. 2C). The endogenous αAmy3 proteins were observed by αAmy3 specific antibodies in wild type (WT), Line-36, and Line-46 cells but were absent in Line-96 cells (Fig. 2C). These results indicate that the mature human BMP2 protein was produced from the artificially intron-inserted *rhBMP2m* recombinant gene controlled by the sugar-regulated endogenous *αAmy3* promoter in rice cell suspension cultures. In a previous study (Nguyen et al. 2022), we reported that the endogenous αAmy3 signal peptide directed a recombinant GFP protein to be secreted outside the cell. To determine whether rhBMP2m protein is secreted into the culture medium of the selected cell lines, we examined suspension cultures of WT, Line-36, Line-46, and Line-96. The αAmy3 proteins were detected in the culture media of WT, Line-36, and Line-46 cell lines (Supplementary Fig. S5A), but not in the *αAmy3SP-rhBMP2m* homozygotic cell line, Line-96. We were also unable to detect the presence of rhBMP2m protein in a ten-fold concentrated sugar-free culture medium for all selected suspension cell lines (Supplementary Fig. S5B). These results suggested that the rhBMP2m recombinant protein was not secreted into the culture medium by these rhBMP2m knock-in cell lines.

### Production of glycosylated and dimerized rhBMP2m was observed in the *rhBMP2m* knock-in cell line

**Fig. 3:**
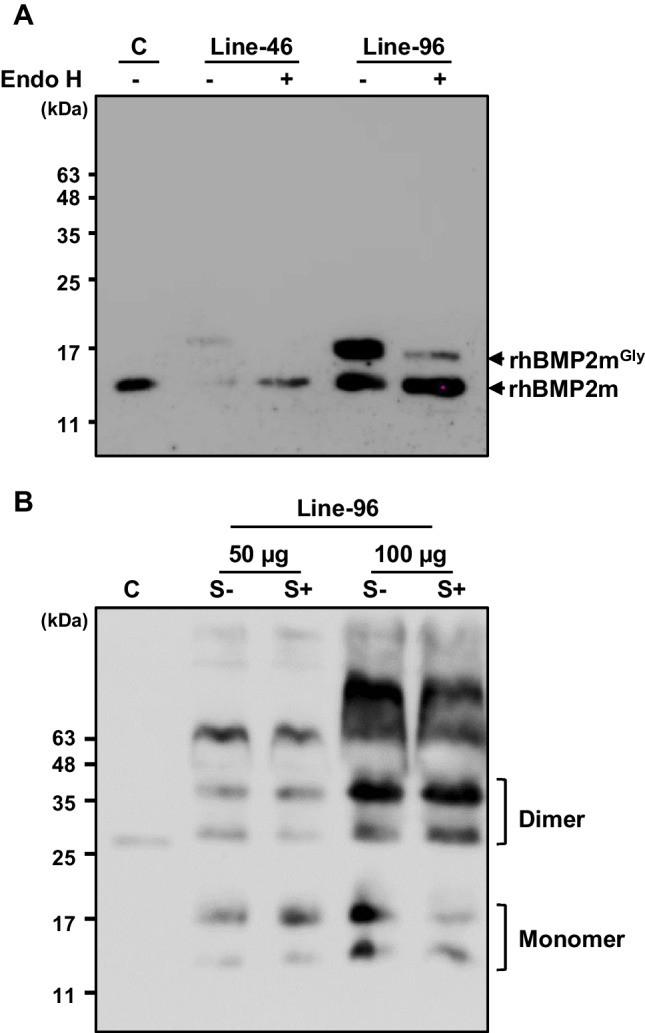
Detection of the glycosylated and dimerized form of rhBMP2m in *rhBMP2m* knock-in rice cell lines. Total soluble proteins were isolated from Lines-46 and -96 after 8 days of culture under sugar-fed (S +) and sugar-starved (S-) conditions. (**A**) Treatment with endoglycosidase of the rhBMP2m proteins. Protein samples from sugar-starved cells were subjected to Endo H endoglycosidase treatment. Subsequently, the presence of rhBMP2m was detected by western blotting with human BMP2 antibodies. The recombinant BMP2 protein (C) from the *E. coli* expression system was used as a control. The symbols “ + ” and “-” represent samples treated with and without the enzyme, respectively. (**B**) The dimeric form of rhBMP2m were identified in a knock-in rice cell line. Total cellular proteins from Line-96 were separated by PAGE without β-mercaptoethanol. Subsequently, the presence of dimeric and monomeric rhBMP2m proteins was detected via western blotting with human BMP2 antibodies. The molecular weight markers, in kDa, are represented on the left side of the figure

The molecular weights of rhBMP2m produced from the sugar-starved cells were approximately 14 and 18 kDa (Fig. 2C). The higher molecular weight of rhBMP2m compared to the 13 kDa molecular weight of commercial *E. coli*-produced hBMP2m suggests that rhBMP2m may undergo post-translational modification. The mature human BMP2 is known to undergo N-glycosylation in CHO cells, and analysis of its amino acid sequence revealed a potential N-glycosylation site. To investigate whether the increased molecular mass of rhBMP2m was attributable to N-glycosylation, total cellular protein from Line-96 cells was treated with Endo H endoglycosidase (Fig. 3A). Remarkably, the levels of rhBMP2m (18 kDa) were reduced to match those of *E. coli*-produced BMP2m (13 kDa) after a 30-min treatment with Endo H (Fig. 3A). These results provided strong evidence that rhBMP2m was N-glycosylated.

Mature human BMP2 is reported to naturally form an active homodimer, with a disulfide bond linking the seventh cysteines of each monomer (Scheufler et al. 1999). To investigate whether rhBMP2m also forms homodimers, total soluble protein (TSP) was isolated from sugar-starved Line-96 cells using β-mercaptoethanol-free protein extraction buffer to keep cysteine dimerization. Subsequently, the proteins were separated by native PAGE and analyzed by western blotting (Fig. 3B). The results revealed the presence of rhBMP2m proteins with molecular weights of 14 and 18 kDa, as well as 28 and 36 kDa rhBMP2m proteins in the cell extracts (Fig. 3B). This result suggests that the 28 and 36 kDa intracellular rhBMP2m proteins obtained from Line-96 exist in the homodimer form.

### The yield of rhBMP2m in the *hBMP2m* knock-in cell line

**Fig. 4:**
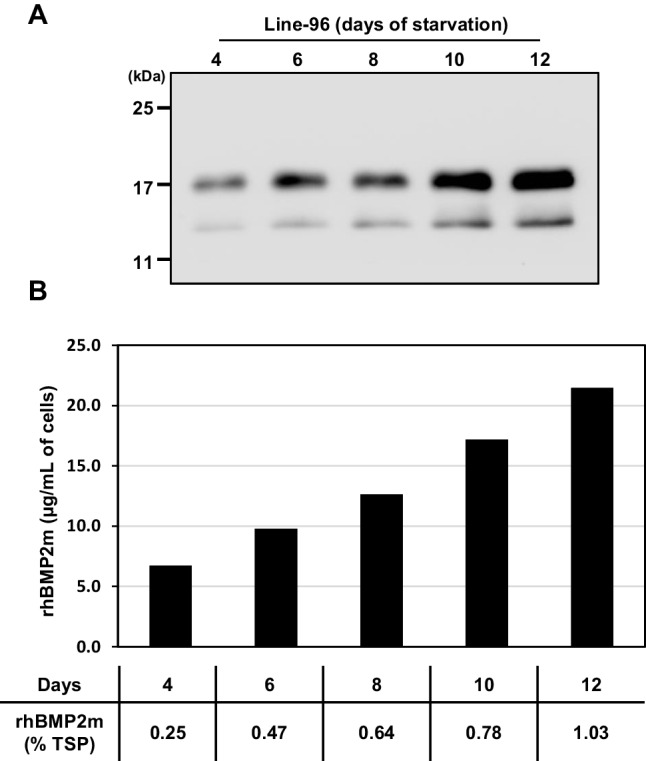
Profiling of rhBMP2m recombinant protein production in suspension cell cultures of Line-96. (**A**) One milliliter of suspension cells was cultured in 2 mL sugar-free N6 medium for various periods. Total cellular soluble protein was isolated in 1 mL protein extraction buffer from each treated cell culture. Equal amounts of total protein from each sample were used for western blotting with human BMP2 antibodies. The molecular weight markers, in kDa, are represented on the left side of the figure. (**B**) Productivity of rhBMP2m in the rice suspension culture. The level of rhBMP2m was determined by ELISA relative to a standard curve based on the amount of *E. coli*-derived hBMP2m protein (upper panel). The lower panel of (**B**) shows the ratio of the rhBMP2m protein to total soluble protein (TSP) for different durations of sugar-starved rice cells

To determine the highest cellular rhBMP2m production yield in the Line-96, 1 mL of cells was cultured in 2 mL sugar-free medium for 4–12 days. The abundance of rhBMP2m during sugar starvation was detected via western blotting using rhBMP2 antibodies. rhBMP2m was initially detected on day 4 (Fig. 4A), and the yield increased until day 12 (Fig. 4A). The protein yield of rhBMP2m was monitored by ELISA. Similar results were obtained by western blotting, the yield of rhBMP2m increasingly progressed from day 4 to a maximum of 21.5 μg/mL of cells on day 12, representing 1.03% of TSP (Fig. 4B).

### Rice-produced rhBMP2m proteins have bioactivity

**Fig. 5:**
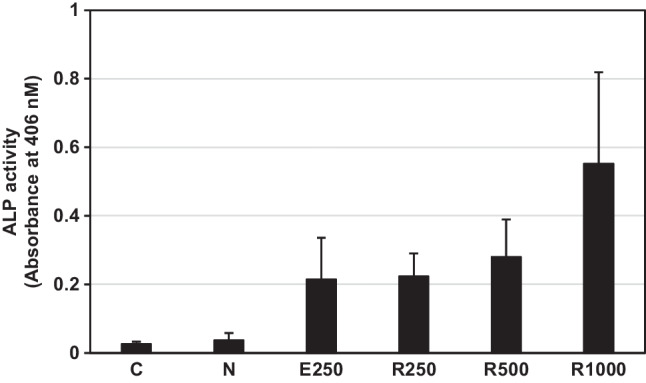
Analysis of the biological activity of secreted rhBMP2m. The C2C12 was incubated with three different concentrations of rhBMP2m: R250 (250 ng/mL), R500 (500 ng/mL), and R1000 (1000 ng/mL). Commercial hBMP2m derived from *E. coli* cells (E250; 250 ng/mL) was used as a reference standard. PBS buffer (C) and non-transformed rice extract (N) were used as negative controls. The ALP activity was measured. The error bar represents the standard deviation from five cultures

The bioactivity of rhBMP2m was determined by measuring the activity of alkaline phosphatase (ALP) induced in C2C12 cells by the mature human BMP2. C2C12 cells were incubated with WT cell extracts, 250 ng/mL *E. coli*-derived hBMP2m (E250), and Line-96 cell extracts with 250 (R250), 500 (R500), and 1000 (R1000) ng/mL rhBMP2m for three days. ALP activity was around 0.02 in C2C12 cells cultured in a medium that contained no recombinant hBMP2m (Fig. 5). In contrast, the ALP activity increased significantly in the presence of rhBMP2m (Fig. 5). This result demonstrates the biological activity of the rhBMP2m protein, which was similar to that of *E. coli*-derived hBMP2 (Fig. 5).

### Production of rhBMP2m proteins from *rhBMP2* knock-in rice T1 seed-derived suspension cultures

**Fig. 6:**
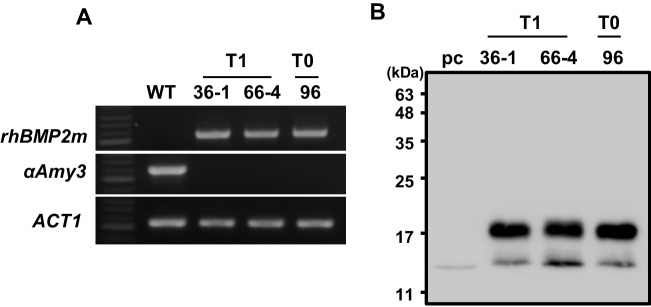
Production of rhBMP2m in the T1 generation of rhBMP2m knock-in rice suspension cell lines. One milliliter of suspension cells, consisting of wild-type (WT), Line-96 (T0) and two homozygotic T1 seed-derived suspension cell lines, 36–1 and 66–4, were cultured in 2 mL sugar-free N6 medium for 8 days. (**A**) Total RNAs were isolated, and RT-PCR was performed using specific primers for *rhBMP2m*, *αAmy3*, and *ACT1* (Table S1). (**B**) Total cellular soluble proteins were also extracted, and equal amounts of total protein from each sample were subjected to western blotting using human BMP2 antibodies. Thirty nanograms of the recombinant BMP2 proteinfrom the *E. coli* was used as a positive control (pc). The molecular weight markers, in kDa, are represented on the left side of the figure

T1 calli were obtained from seeds of three T1 transgenic lines, Lines 1–10, 1–36, and 1–66. PCR-based genotype analysis showed that Lines T1-36–1, T1-36–4, T1-66–3, and T1-66–4 were homozygous, each harboring *αAmy3sp-BMP2* recombinant DNA and lacking the endogenous *αAmy3* genes (supplementary Fig. S6). To evaluate their potential for high-yield expression of *rhBMP2m*, suspension cell lines were established from T1-36–1 and T1-66–4. Total RNAs and proteins were isolated from these lines following eight days of sugar starvation. RT-PCR analysis using specific primer sets confirmed the presence of *rhBMP2m* mRNAs in both sugar-starved cell lines (Fig. 6A). Furthermore, western blot analysis using anti-hBMP2 antibodies detected rhBMP2m in both sugar-starved cell lines (Fig. 6B). In addition, the yield of rhBMP2m was comparable to that of the T0 homozygotic suspension cell line, Line-96 (Fig. 6B), indicating the genetic stability of *rhBMP2m* gene knock-in at *αAmy3* intron I and its consistent expression across two generations of rice suspension cells.

## Discussion

We successfully produced a bioactive pharmaceutical protein, the mature human BMP2, in rice suspension cell cultures. Using the modified CRISPR/Cas 9 intron-targeted insertion strategy, we inserted the rice-codon optimized mature human BMP2 cDNA (*rhBMP2m*) directly into the first intron of *αAmy3* gene. Our results demonstrated that 13.5% of transformed rice calli carried the correct orientation of *rhBMP2m* DNA, consistent with previous studies (Nguyen et al. 2022) in which the *GFP* gene was inserted into the *αAmy3* intron 1 using the same knock-in technique. The native promoter of *αAmy3* effectively regulated *rhBMP2m* expression under sugar-deficient conditions. The rhBMP2m recombinant protein yielded 21.5 μg/mL of cells, equivalent to 1.03% of the TSP.

To date, to the best of our knowledge, the successful expression of the mature form of human BMP2 using plant systems has not been reported. Previous attempts to introduce *hBMP2m* DNA into the lettuce chloroplast genome did not result in significant accumulation of hBMP2m (Queiroz et al. 2019). Other studies produced hBMP2m by fusing it with GUS and zein at the N-terminus, but the hBMP2m recombinant protein yield in tobacco leaves dropped to a mere 0.02% TSP after removing the fused domains (Ceresoli et al. 2016; Suo et al. 2006). Our recent work surpasses these previous achievements (Ceresoli et al. 2016; Suo et al. 2006), highlighting the effectiveness of our approach for generating higher yields of mature human BMP2.

A correlation between gene dosage and expression level has been observed, wherein an increase in gene number generally enhances gene expression (Hood and Requesens 2012). Homozygous *rhBMP2m* knock-in suspension cell cultures exhibited a higher yield of rhBMP2m recombinant protein, indicating the influence of gene dosage on expression levels. Therefore, using homozygous *rhBMP2m* knock-in suspension cell cultures is a viable approach to enhance rhBMP2m recombinant protein production. We successfully obtained a homozygous *rhBMP2m* knock-in cell line at a frequency of 1% from the T0 transformants. However, in our previous study (Nguyen et al. 2022), no homozygous *GFP* knock-in cell lines were found among 217 potential transformants, suggesting that the actual frequency of generating homozygous knock-in cell lines from the T0 generation using this approach might be less than 1%. Alternatively, homozygous *rhBMP2m* knock-in suspension cultures could be generated from the T1 seed population. The high rhBMP2m recombinant protein yield observed in T1 seed-derived knock-in suspension cultures further supports this effective means of achieving gene dosage.

The αAmy3 signal peptide has been previously reported to effectively direct cargo proteins for secretion from cells (Chen et al. 2004). Various recombinant proteins have been successfully secreted into the liquid culture media using the αAmy3 signal peptide (Hong et al. 2006; Huang et al. 2005, 2021, 2015; Liu et al. 2015; Nam et al. 2017; Sinaga et al. 2021; Van Giap et al. 2019). However, in this study, the endogenous αAmy3 signal peptide failed to direct the rhBMP2m recombinant protein from the selected *rhBMP2m* knock-in suspension cultures into the culture medium. This contrasts with a previous study, in which the GFP recombinant protein was successfully secreted into the culture medium using the native αAmy3 signal peptide (Nguyen et al. 2022). This difference may be attributed to the specific characteristics of human BMP2 protein. In human cells, BMP2 is synthesized as pre-pro-BMP2 and undergoes processing to produce an active hBMP2m homodimer (Wozney et al. 1988). Although the propeptide of hBMP2 is not required for biological activity (Wang et al. 1990), it plays a role in the secretion of the mature hBMP2 (Israel et al. 1992). Studies conducted with CHO cells have shown that deletion of the hBMP2 propeptide prevents the secretion of the hBMP2m recombinant protein (Israel et al. 1992). Additionally, mutation of the N135Q-glycosylation site within the hBMP2 propeptide hinders efficient secretion by CHO cells, resulting in the intracellular accumulation of hBMP2 (Hang et al. 2014). These findings indicate that post-translational processes involving the hBMP2 propeptide are crucial for the secretion of the mature hBMP2. In this study, wherein only the mature hBMP2 was expressed, the lack of post-translational processing of the propeptide may have hindered the ability of αAmy3 signal peptide to secrete rhBMP2m from the rice suspension cells. Another approach involves fusing the γ-zein N-terminal domain to the mature hBMP2 to accumulate zein-hBMP2m protein in protein bodies; however, zein-hBMP2m accumulates in the endoplasmic reticulum (ER) and cannot target the protein bodies (Ceresoli et al. 2016). These studies further support the idea that specific processes involving the hBMP2 propeptide are crucial for determining the targeting and secretion of the mature human BMP2.

The mature form of human BMP2 is a secreted glycoprotein that acquires N-glycans during trafficking through the ER and Golgi complex. In CHO cells, the secreted hBMP2m protein containing N-glycans is sensitive to endoglycosidase H (Hang et al. 2014). In this study, it was discovered that rhBMP2m, produced by rice suspension cells, is a glycosylated protein, and glycans attached to rhBMP2m can be removed by endoglycosidase H. This suggests that rhBMP2m undergoes trafficking to the ER, which is facilitated by the fusion of the αAmy3 signal peptide at its N-terminus. However, whether rhBMP2m also traffics through the Golgi complex in rice cells remains unclear. In CHO cells, an N135Q-glycosylation site mutant of hBMP2 affects its secretion and leads to its retention in the ER (Hang et al. 2014). Similarly, the zein-hBMP2m fusion protein accumulates in the ER of tobacco cells (Ceresoli et al. 2016). Therefore, it is reasonable to speculate that rhBMP2m produced in rice cells accumulates within the ER. However, further investigations are required to precisely determine the localization of rhBMP2m in rice cells. Such studies will contribute to our understanding of the αAmy3 signal peptide-based recombinant protein secretion system in rice cell cultures.

## Supplementary information

Below is the link to the electronic supplementary material.

Supplementary file1 (PDF 21284 KB)

## Data Availability

All data generated or analyzed during this study are included in this published article and its supplementary information files.
