# Supplementary material for: High-yield BMP2 expression in rice cells via CRISPR and endogenous αAmy3 promoter
Source: Appl Microbiol Biotechnol. 2024 Feb 14;108(1):206. doi: 10.1007/s00253-024-13054-0 (PMC10867061; doi:10.1007/s00253-024-13054-0)
Supplement: Supplementary file 1 — Supplementary file1 (PDF 21284 KB) [file 253_2024_13054_MOESM1_ESM.pdf]

Applied Microbiology and Biotechnology

Supplementary Information

High-Yield BMP2 Expression in Rice Cells *via* CRISPR and Endogenous *αAmy3* Promoter

Thi Mai Nguyen[1,2], Pei-Yi Wu[2], Chih-Hung Chang[1, 3], Li-Fen Huang[1]*

[1]Graduate School of Biotechnology and Bioengineering, Yuan Ze University, Taoyuan City 320, Taiwan, ROC.

[2]Department of Life Sciences, National Central University, Taoyuan City 320, Taiwan, ROC.

[3]Department of Orthopedic Surgery, Far Eastern Memorial Hospital, New Taipei City, Taiwan, ROC.

*Corresponding author: Li-Fen Huang, Orcid-ID: 0000-0002-7484-4148, Tel: 886-3-4338800 ext 2189; Fax: 886-3-4334667; E-mail: hlf326@saturn.yzu.edu.tw

Supplementary Table 1. Primer details and amplifications for PCR

**Table 1**	**Primer details and amplifications for PCR**			
Gene	Primer sequences (5′–3′, forward, reverse)	Ta (C)	Product size (bp)	Target
Cas9F<br>Cas9R	TATGGCGGCTTCGATTCTCCG<br>GGTAGAGGAAGTTCACGTAC	55	316	*Cas9*
rBMP2F<br>rBMP2R	GATTGAATCCTGTTGCCGGTCTTGCG<br>CACCCACGTCTGGCTGCCGCCGCCG	55	364	*rhBMP2m*
Act1F<br>Act1R	AGACCTTCAACACCCCTGCTA<br>CAGGGCGATGTAGGAAAGCTT	55	500	*ACT1*
E1F<br>NOSR	ATGAAGAACACCAGCAGCTTGTG<br>ACATGTTAATTATTACATGCTTAA	50	~829	*αAmy3-rhBMP2m*
E1F<br>rBMP2R	ATGAAGAACACCAGCAGCTTGTG<br>GCGGCCGCATGAGTAAAGGAGAAGAAC	50	~600	*αAmy3-rhBMP2m*<br>(5’ junction)
NOSF<br>E2R4	ACTAGGATAAATTATCGCGCGCGGTGTC<br>AGTACTGCGTGTCGTCGCTGCAGATCATG	55	~450	*αAmy3-rhBMP2m*<br>(3’ junction)
E1F<br>E2R4	ATGAAGAACACCAGCAGCTTGTG<br>AGTACTGCGTGTCGTCGCTGCAGATCATG	50	~600	*αAmy3*

Supplementary Fig. S1

A

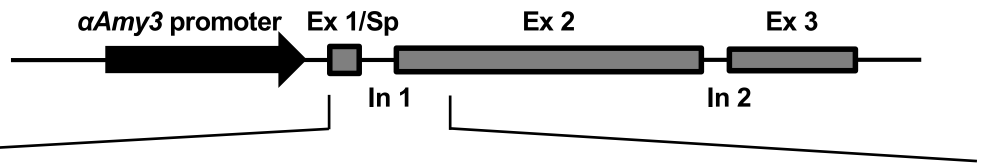


```
ATG AAG AAC ACC AGC AGC TTG TGT TTG CTG CTC CTC GTG GTG CTC TGC AGC TTG ACC TGT AAC
 M   K   N   T   S   S   L   C   L   L   L   L   V   V   L   C   S   L   T   C   N
TCG GGT CAA GCA CAG GTC CTC TTC CAG gtacgtagtactctactacccatcactttctgtgaagactttgctg
 S   G   Q   A   Q   V   L   F   Q
aagaaacacattagaatttgagatatttatgtgtgatcgattgatcacttacctacttataacatgcatcatgcag GGT TTC
                                                                               G   F
AAC TGG GAG TCG TGG AAG CAG CAG GGT GGC TGG TAC AAC ATG TTG AAA GGC CAA
 N   W   E   S   W   K   Q   Q   G   G   W   Y   N   M   L   K   G   Q
```

B

Codon optimized for rice of the mature form of human BMP2 cDNA

```
CAG GCC AAG CAC AAG CAG AGG AAG AGG CTC AAG AGC AGC TGC AAG AGG CAC CCG CTC TAC GTG
 Q   A   K   H   K   Q   R   K   R   L   K   S   S   C   K   R   H   P   L   Y   V
GAC TTC TCC GAC GTG GGC TGG AAC GAC TGG ATC GTG GCC CCG CCG GGC TAC CAC GCC TTC TAC
 D   F   S   D   V   G   W   N   D   W   I   V   A   P   P   G   Y   H   A   F   Y
TGC CAC GGC GAG TGC CCG TTC CCG CTC GCC GAC CAC CTC AAC TCC ACC AAC CAC GCC ATC GTG
 C   H   G   E   C   P   F   P   L   A   D   H   L   N   S   T   N   H   A   I   V
CAG ACC CTC GTG AAC AGC GTG AAC TCC AAG ATC CCG AAG GCC TGC TGC GTG CCG ACC GAG CTC
 Q   T   L   V   N   S   V   N   S   K   I   P   K   A   C   C   V   P   T   E   L
AGC GCC ATC TCC ATG CTC TAC CTC GAC GAG AAC GAG AAG GTG GTG CTC AAG AAC TAC CAG GAC
 S   A   I   S   M   L   Y   L   D   E   N   E   K   V   V   L   K   N   Y   Q   D
ATG GTG GTG GAG GGC TGC GGC TGC AGG TAA
 M   V   V   E   G   C   G   C   R   -
```

C

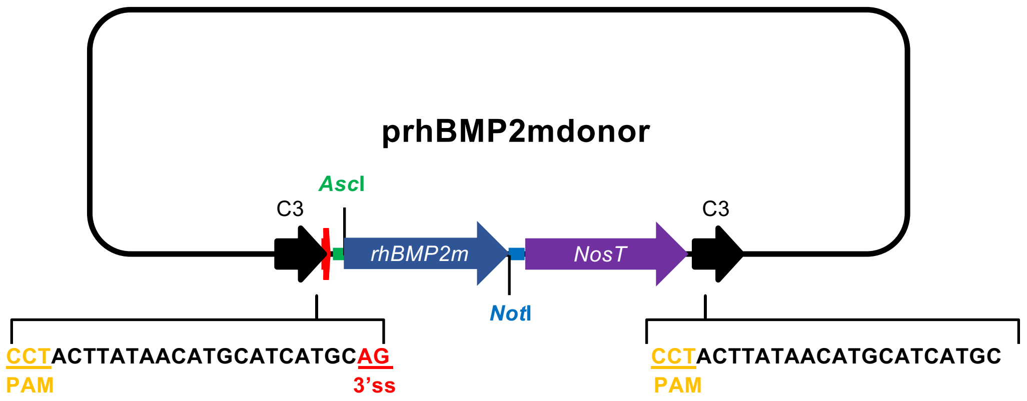

Fig. S1. Diagram illustrating the DNA sequences of the rice *αAmy3* gene, the rice-optimized human mature *BMP2* gene, *rhBMP2m*, and donor construct for CRISPR/Cas9-mediated insertion within *αAmy3* intron 1. (A) Represents the DNA and corresponding amino acid sequences of the rice *αAmy3* gene. This includes exon 1 (Ex 1) encoding the signal peptide (Sp), intron 1 (In), and part of exon 2. The C3 CRISPR target site within intron 1 is underlined to indicate its location. (B) DNA sequence of the rice-optimized human *BMP2* gene is presented with the corresponding amino acids displayed below the sequence. (B) Diagram of the *rhBMP2m* donor plasmid used in this study. The plasmid contains the C3 guide RNA target sites, rice-optimized human mature form of BMP2 gene (*rhBMP2m*), *NosT* terminator, and an artificial 3′ splicing site (3′ss) along with the C3 target site.

Supplementary Fig. S2

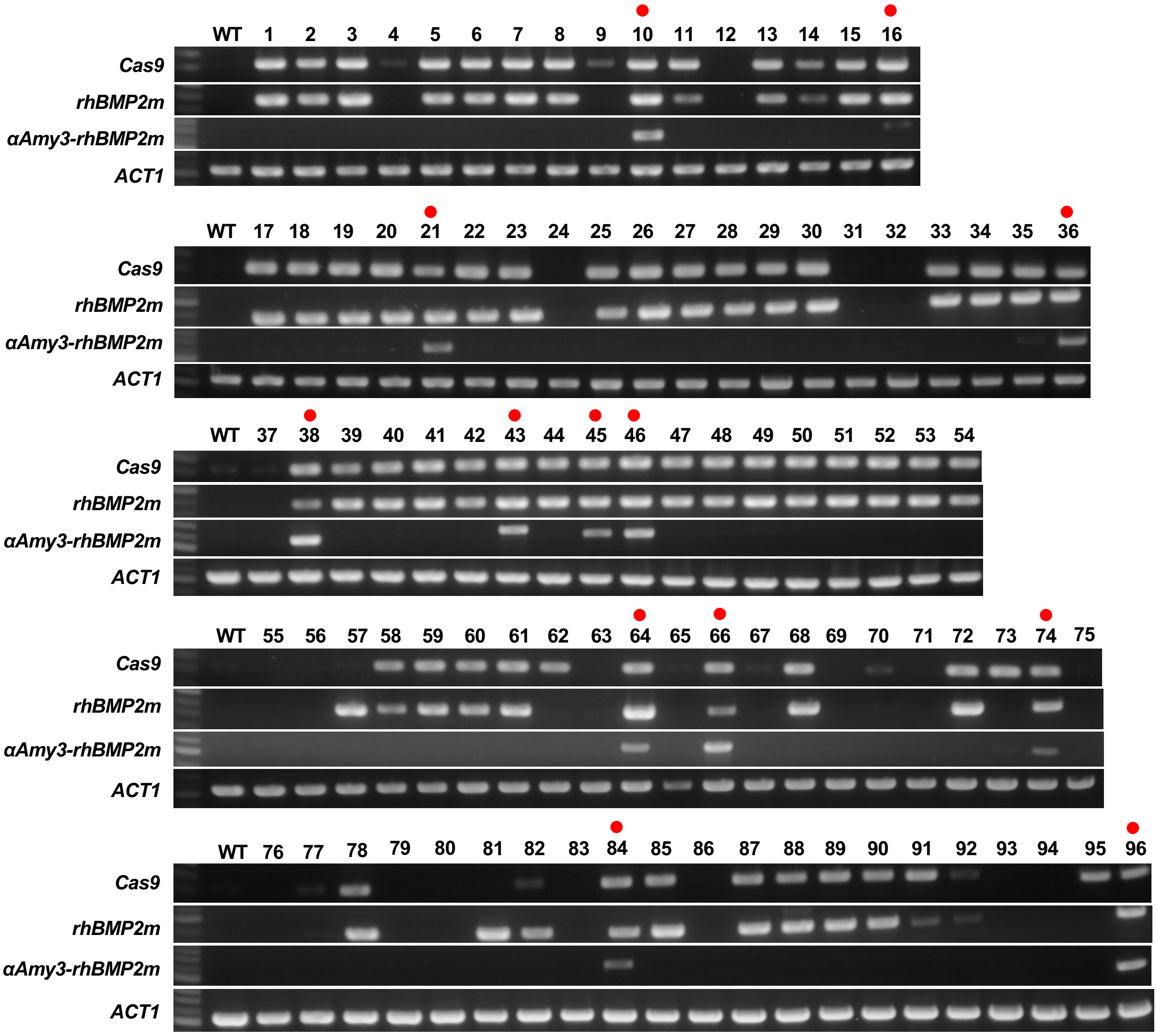

Fig. S2. Genotype analysis of *rhBMP2m* knock-in rice cultured cells. Genomic DNA was extracted from various putative transgenic cell lines and subjected to PCR using specific primers (Supplementary Table 1) targeting *Cas9*, *rhBMP2m*, *αAmy3-rhBMP2m*, and *ACT1*. Red dots indicate putative lines harboring the *rhBMP2m* gene insertion within αAmy3 intron 1.

Supplementary Fig. S3

C3 site 5' junction

WT: CCTACTTATAACATGCATCATGC

Line 1-10: 1 bp insertion
CCTACTATATAACATGCATCATGCAGGGCGCGCCACAGGCCAAGCACAAGCAG

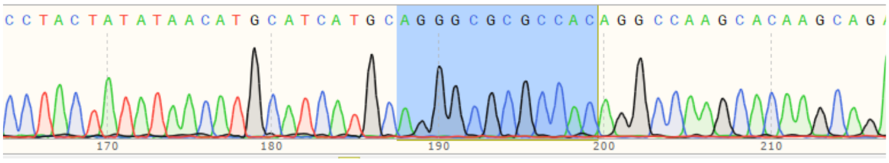

Line 1-16: 122 bp insertion
CCTACTC...TTATAACATGCATCATGCAGGGCGCGCCACAGGCCAAGCACAAGCAG

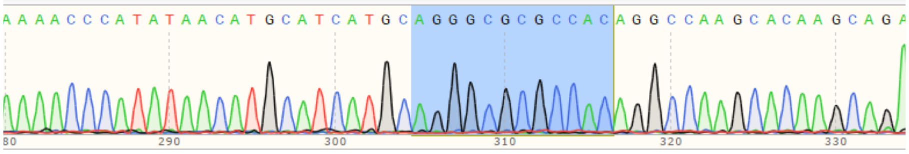

Line 1-36: 13 bp deletion
-------------ATGCATCATGCAGGGCGCGCCACAGGCCAAGCACAAGCAG

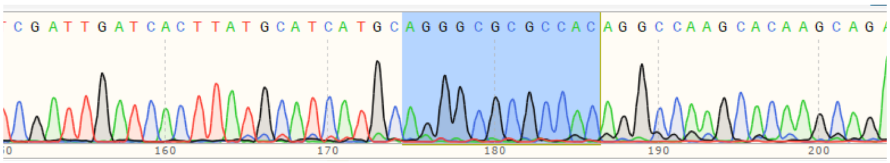

Line 1-46: 1 bp deletion
CCTAC-TATAACATGCATCATGCAGGGCGCGCCACAGGCCAAGCACAAGCAG

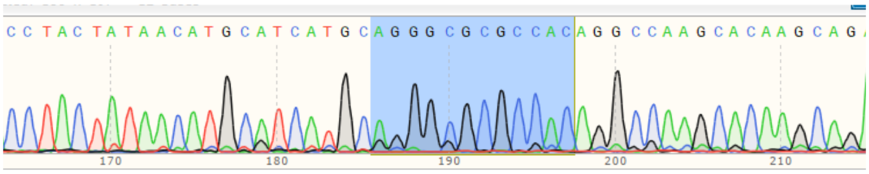

Line 1-66: 28 bp deletion
-...-ATATAACATGCATCATGCAGGGCGCGCCACAGGCCAAGCACAAGCAG

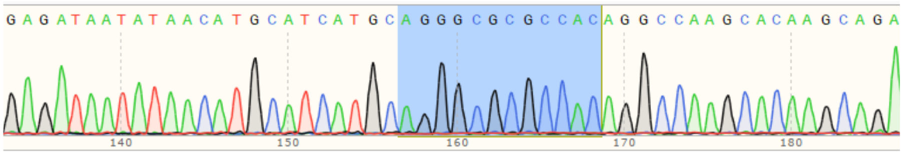

Line 1-96 1 bp deletion
CCTAC-TATAACATGCATCATGCAGGGCGCGCCACAGGCCAAGCACAAGCAG

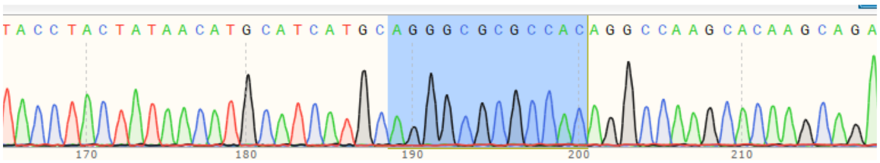

Fig. S3. DNA Sequencing Analysis of 5′- junctions in *mhBMP2m* knock-in transgenic lines. DNA fragments at the 5′-junctions were amplified using PCR with E1F and rhBMP2R primers. The amplified DNA fragments were subsequently subjected to DNA sequencing to confirm the integrity of the junctions and the fidelity of the knock-in sequences.

Supplementary Fig. S4

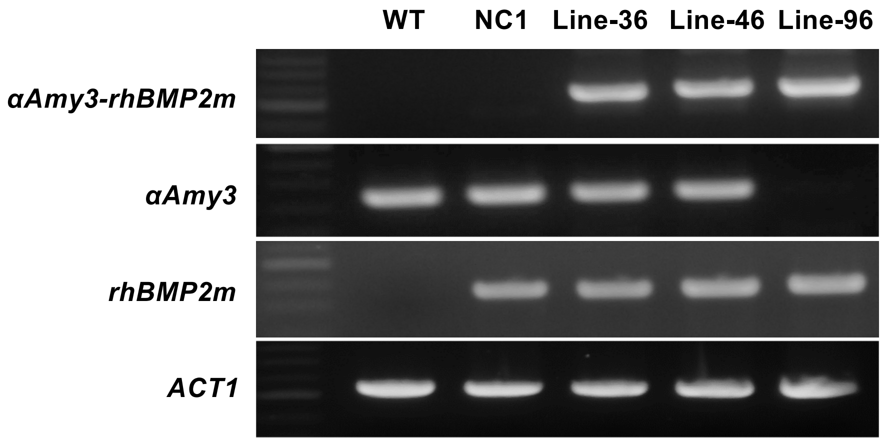

Fig. S4. Genotype analysis of rhBMP2m knock-in rice cell lines. Genomic DNA was extracted from the wild type (WT), one *rhBMP2m* random insertion cell line (NC1), and three knock-in suspension cell lines (Line-36, Line-46, and Line-96). PCR amplification was performed on the extracted genomic DNA using specific primers (Supplementary Table 1) targeting *αAmy3-rhBMP2m*, *αAmy3, rhBMP2m*, and *ACT1*.

Supplementary Fig. S5

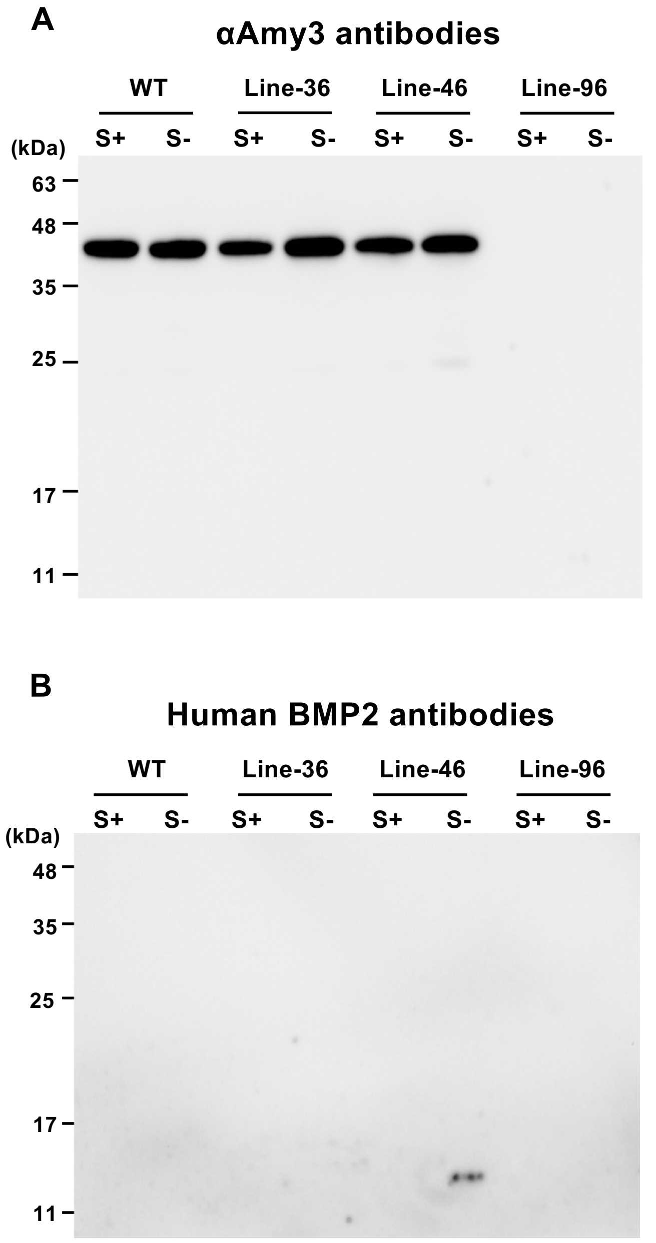

Fig. S5. Detection of rhBMP2m protein in the cultured medium of knock-in rice cell lines. (A) Suspension cells of the wild type (WT) and three knock-in transgenic lines (Line-36, Line-46, and Line-96) were cultured in sugar-supplemented (S+) or sugar-free (S-) medium for 10 days. The collected culture medium was subjected to western blot analysis using αAmy3 antibodies. (B) The culture medium was then condensed 10-fold, and the condensed medium from each cell line was collected and subjected to western blot analysis using human BMP2 antibodies. Numbers on the right indicate the positions of molecular mass markers, in kDa.

Supplementary Fig. S6

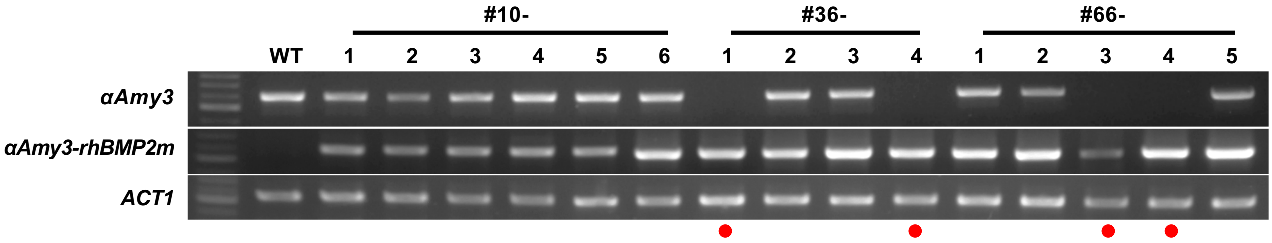

Fig. S6. Genotype analysis of *rhBMP2* knock-in rice T1 seed-derived calli. Genomic DNA was extracted from various calli, originating from T1 seed populations of Line-10, Line-36, and Line-66 and was subjected to PCR using *αAmy3, αAmy3-rhBMP2m*, and *ACT1* specific primers (Supplementary Table 1), respectively. Red dots in the figure indicate homozygotes.
